# Atelectasis secondary to two separate video-assisted thoracoscopic wedge resection of the lung lobe and pleurodesis under general anesthesia in the same patient: a case report

**DOI:** 10.3389/fped.2026.1796447

**Published:** 2026-09-07

**Authors:** Jin Yuan Wang, Yu Li

**Affiliations:** Department of Anesthesiology, Women and Children's Medical Center (Group), Dalian, China

**Keywords:** atelectasis, general anesthesia, mucus sputum, pneumothorax, video-assisted thoracoscopic wedge resections of the lung lobe and pleurodesis

## Abstract

**Background:**

Intraoperative atelectasis is a common pulmonary complication during thoracic surgery, yet recurrent mucus-mediated atelectasis during lung re-expansion in the same patient undergoing sequential two video-assisted thoracoscopic wedge resections of the lung lobe and pleurodesis successively is extremely rare.

**Case description:**

This report describes a 15-year-old previously healthy adolescent who developed recurrent intraoperative atelectasis secondary to extensive viscous mucus plug obstruction during two successive video-assisted thoracoscopic wedge resections and pleurodesis for recurrent spontaneous pneumothorax. Notably, the patient presented no preoperative respiratory symptoms before either operation but exhibited persistent subclinical laboratory inflammatory abnormalities secondary to prior mycoplasma pneumoniae and human rhinovirus infections. Adaptive real-time video-assisted airway surveillance was implemented in the second surgery to dynamically monitor intraoperative mucus formation, enabling timely bronchoscopic lavage and mucus clearance. Atelectasis following thoracic surgery is a common pulmonary complication; however, it is rare for the same patient to experience atelectasis due to airway obstruction by copious, viscous sputum generated during surgery on two separate operation.

**Conclusions:**

Our findings indicate that persistent subclinical airway inflammation induced by prior respiratory infections, combined with surgical stress and one-lung ventilation (OLV)-related pulmonary inflammatory injury, synergistically triggers intraoperative airway mucus hypersecretion, resulting in recurrent obstructive atelectasis. This case highlights the clinical necessity of comprehensive preoperative evaluation of subclinical inflammatory status and individualized intraoperative airway management particularly the utilization of visualized airway monitoring devices for adolescent patients undergoing thoracic surgery.

## Introduction

Primary spontaneous pneumothorax (PSP) predominantly affects adolescents and young adults without preexisting clinically apparent pulmonary parenchymal diseases (1). First-line management for initial PSP includes observational therapy, needle aspiration, and closed thoracic drainage. For patients with persistent air leakage lasting more than 5–7 days or failed lung re-expansion after conservative treatment, surgical intervention via video-assisted thoracoscopic surgery (VATS) is strongly recommended.

Protective lung ventilation strategies are routinely implemented in patients undergoing VATS to optimize airway management and mitigate postoperative pulmonary complications. As the most prevalent pulmonary complication following thoracic surgery, atelectasis significantly impairs perioperative oxygenation and adversely affects postoperative functional recovery. Usually in VATS procedures, anesthesiologists collaborate closely with surgeons to conduct intraoperative lung recruitment maneuvers and airway secretion clearance. This multimodal intraoperative intervention effectively restores alveolar gas exchange, maintains stable perioperative oxygenation, and lowers the risk of postoperative pulmonary complications.

This case report describes a 15-year-old female patient who underwent two sequential video-assisted thoracoscopic wedge resections of the lung lobe and pleurodesis for recurrent unilateral spontaneous pneumothorax. The patient developed identical intraoperative atelectasis caused by extensive viscous mucus plug obstruction during lung re-expansion in both operations. Real-time video-assisted airway surveillance was applied in the second surgery to dynamically track intraoperative airway changes and mucus generation. The rarity of this recurrent perioperative complication provides valuable clinical insights for optimizing perioperative airway assessment and anesthetic management in adolescent thoracic surgery patients.

## Case presentation

### Patient history and preoperative evaluation

A 15-year-old female adolescent (height 170 cm, weight 53 kg, BMI 18.3 kg/m²) was admitted to our thoracic surgery department on April 29, 2024, presenting with half a day of right-sided thoracodynia and chest tightness. Six months prior to admission, the patient suffered from fever and cough caused by Mycoplasma pneumoniae infection, although chest CT at that time showed no definite pulmonary consolidation or pneumonia lesions. Twenty days before the first admission, she received closed thoracic drainage for right spontaneous pneumothorax and achieved symptomatic relief before discharge.

**Figure 1:**
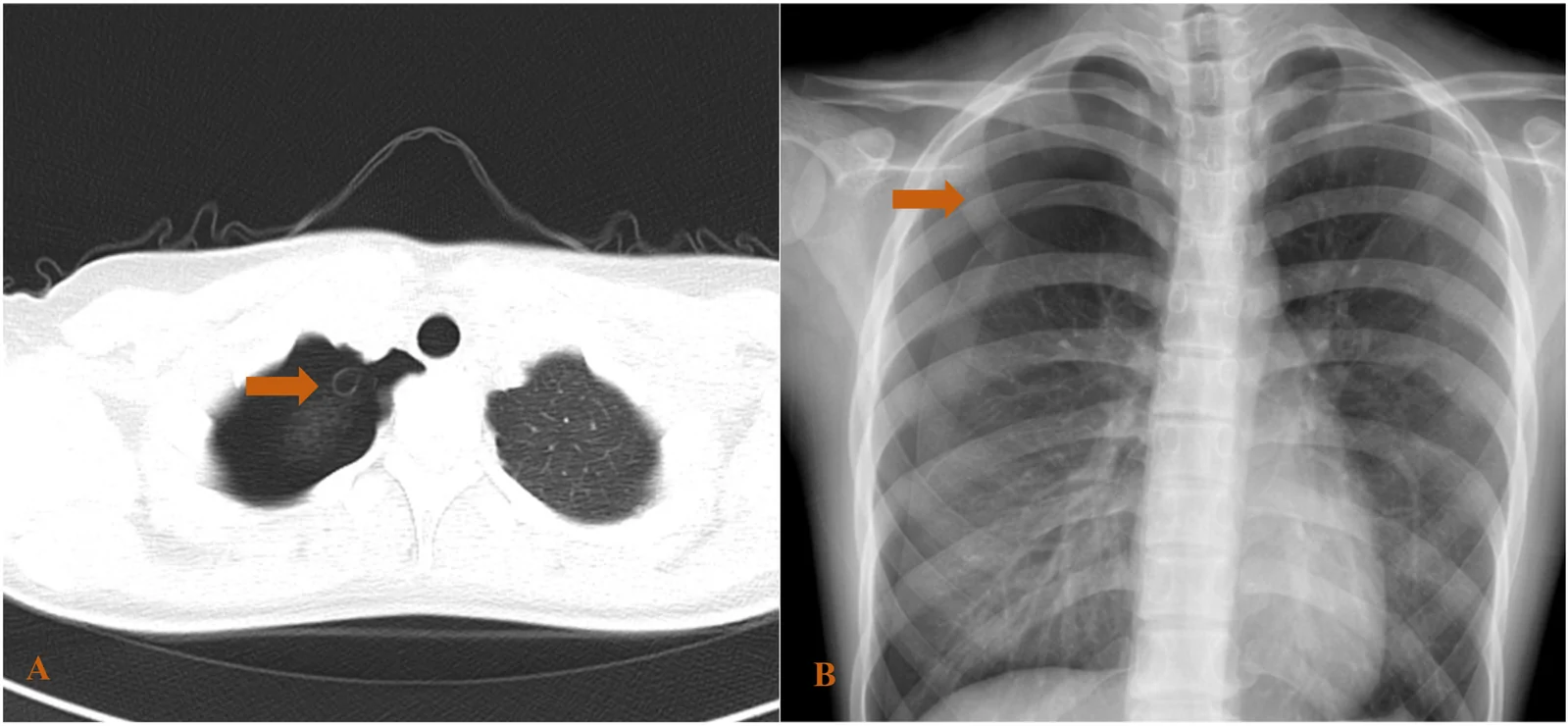
Preoperative chest imaging of the first surgery. **(A)** Chest CT showing right apical pulmonary bullae; **(B)** Chest x-ray demonstrating right spontaneous pneumothorax.

Upon current admission, chest CT confirmed right-sided pneumothorax and subpleural bullae at the right lung apex (Figure 1A: Chest CT showing right pulmonary bullae; Figure 1B: Chest x-ray confirming right pneumothorax). The patient was diagnosed with recurrent right spontaneous pneumothorax. Preoperative routine blood tests, coagulation function, and biochemical examinations revealed no overt abnormalities. Preoperative vital signs were stable: blood pressure 107/58 mmHg, heart rate 82 beats/min, and peripheral oxygen saturation 100% on room air.

### First surgical procedure (right video-assisted thoracoscopic wedge resections of the lung lobe and pleurodesis)

Emergency video-assisted thoracoscopic wedge resections of the right lung lobe and pleurodesis were performed under general anesthesia. Anesthesia induction was conducted with midazolam 4 mg, sufentanil 20 µg, etomidate 15 mg, and rocuronium 30 mg, with normal mask ventilation and lung compliance confirmed. A 7.0-sized reinforced endotracheal tube was inserted under video laryngoscopy, followed by placement of a 9F bronchial blocker into the right main bronchus under flexible bronchoscopic guidance. Ultrasound-guided arterial and venous catheterization was completed for intraoperative hemodynamic monitoring.

Intraoperative anesthesia was maintained with combined intravenous and inhalation anesthesia. At the initiation of surgery, standard mechanical ventilation parameters were set as follows: oxygen flow 3 L/min, fractional inspired oxygen 40%–100%, tidal volume 7 mL/kg, respiratory rate 15 breaths/min, and inspiratory-to-expiratory ratio 1:2 during bilateral lung ventilation. Continuous intraoperative monitoring included end-tidal carbon dioxide, peak and plateau airway pressure, airway resistance, invasive arterial pressure, central venous pressure, entropy index, and body temperature, with arterial blood gas analysis performed hourly. Ten minutes after surgical initiation, OLV was established with adjusted ventilation parameters: tidal volume 6 mL/kg, respiratory rate 18 breaths/min, and inspiratory-to-expiratory ratio 1:2. All intraoperative monitoring indicators remained stable throughout the procedure.

At 1 h and 58 min after surgery commencement, right upper lobe wedge resection was completed. Intraoperative lung inflation was performed to detect air leakage, followed by conversion from OLV to bilateral lung ventilation. Repeated manual lung inflation failed to resolve persistent partial atelectasis of the right upper lobe, and routine airway suction achieved no improvement.

Flexible bronchoscopy exploration revealed complete obstruction of the right upper lobe bronchial orifice by thick, gelatinous mucus, which was inaccessible to conventional suction catheters. Given the limited suction and lavage function of the intraoperative portable bronchoscope, multidisciplinary consultation with the pulmonology department was conducted after full communication with the patient's guardians. Electronic fiberoptic bronchoscopy and targeted airway lavage were subsequently performed.

**Figure 2:**
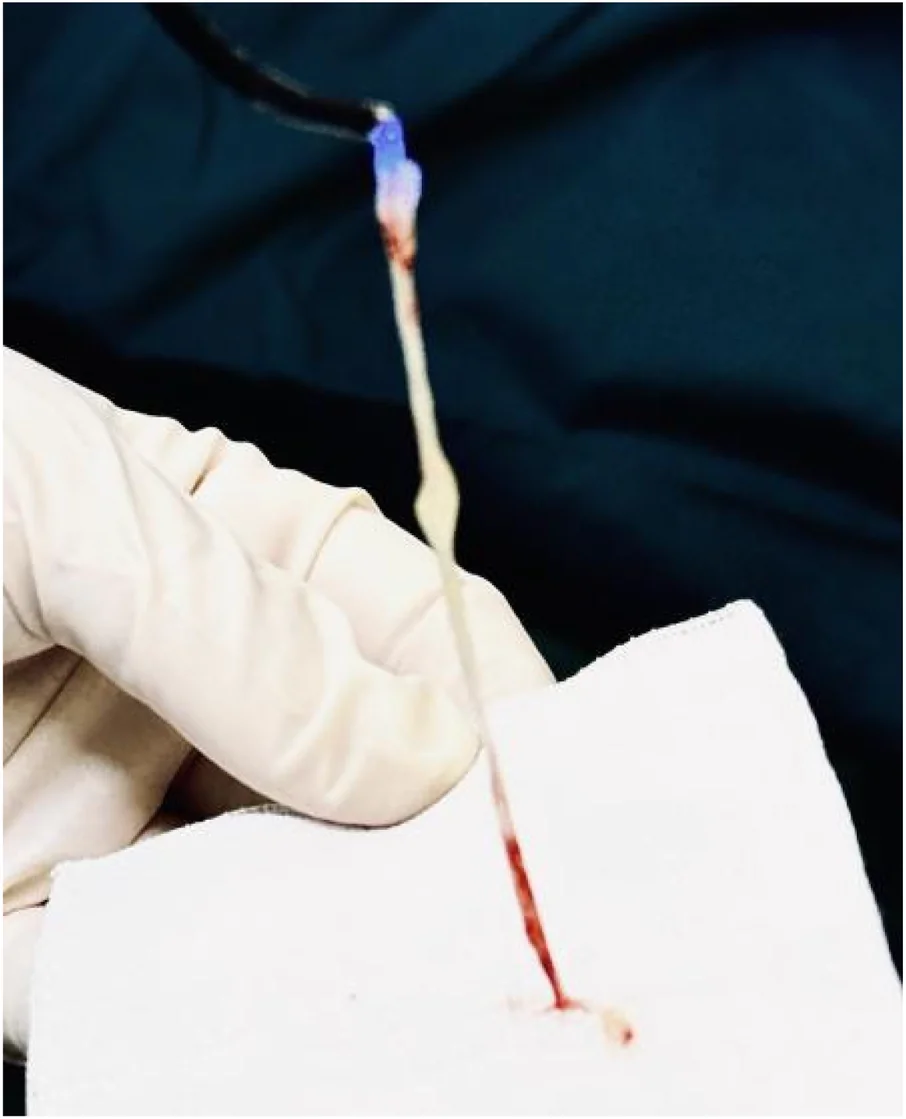
Bronchoscopic view after airway lavage, showing complete clearance of viscous mucus plugs in the right upper lobe bronchus.

Detailed bronchoscopic examination demonstrated extensive thick white mucus plugs obstructing the segmental orifices of the right upper lobe, right middle lobe, right lower lobe basal and dorsal segments, as well as the lingular and proper segments of the left upper lobe. After repeated normal saline lavage and thorough mucus clearance, satisfactory re-expansion of all lung segments was achieved (Figure 2: Bronchoscopic view showing cleared viscous sputum after lavage). The thoracic drainage tube was placed, and the operation was successfully completed.

Intraoperative vital signs remained stable. The total operative duration was 4 h and 40 min. Twenty-five minutes postoperatively, the patient recovered satisfactory spontaneous respiration with a modified Aldrete score of 8, and tracheal extubation was performed. The patient was returned to the ward in stable condition.

Postoperative chest x-ray on day 4 showed complete resolution of pneumothorax. Pathological examination of resected right lung tissue revealed alveolar fusion, extensive chronic inflammatory cell and foam cell infiltration, and marked vascular congestion, consistent with pulmonary bullae formation. The patient was discharged uneventfully on postoperative day 6.

### Second surgical procedure (left video-assisted thoracoscopic wedge resections of the lung lobe and pleurodesis)

**Figure 3:**
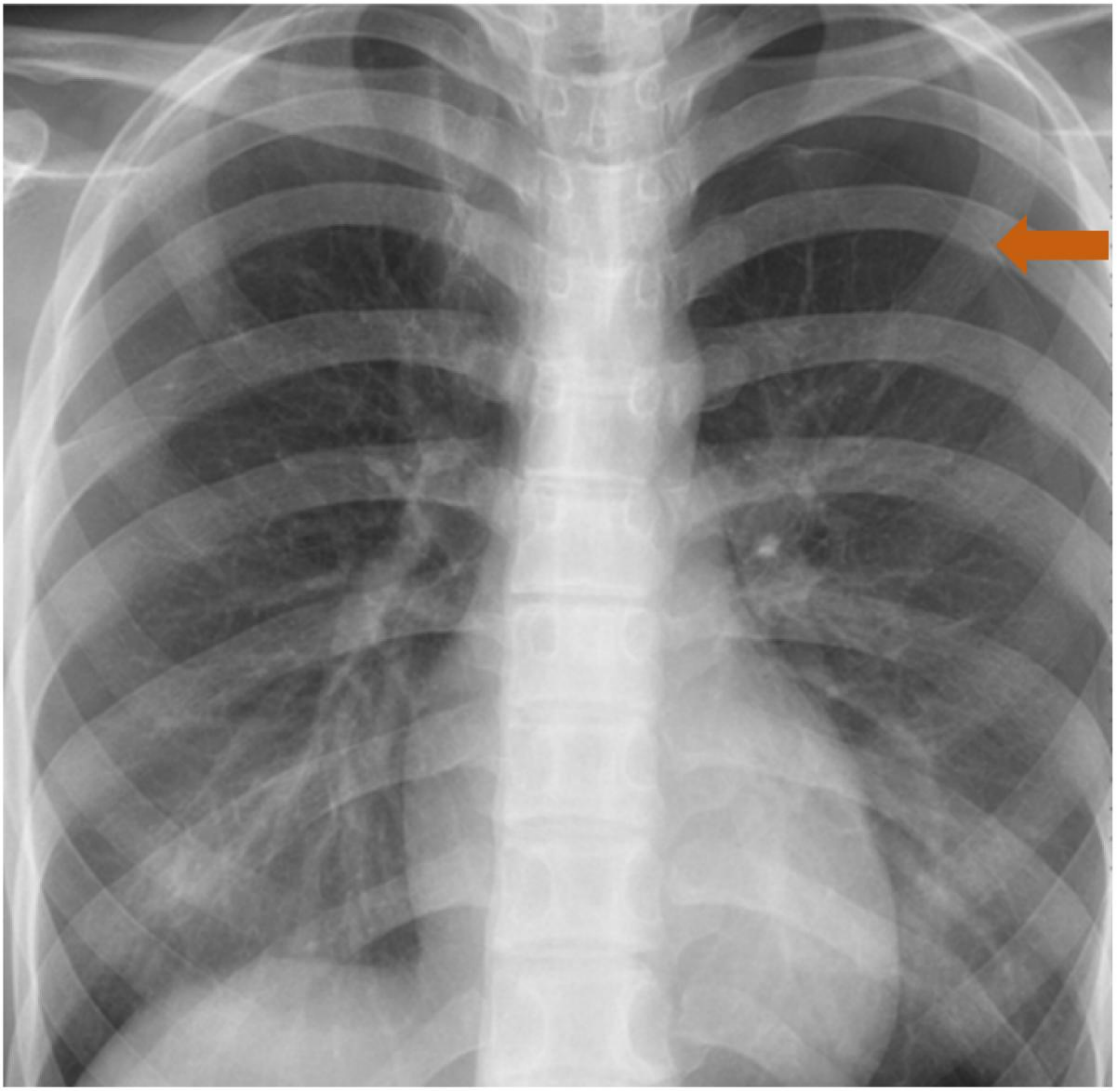
Chest x-ray before the second surgery, confirming left spontaneous pneumothorax.

**Figure 4:**
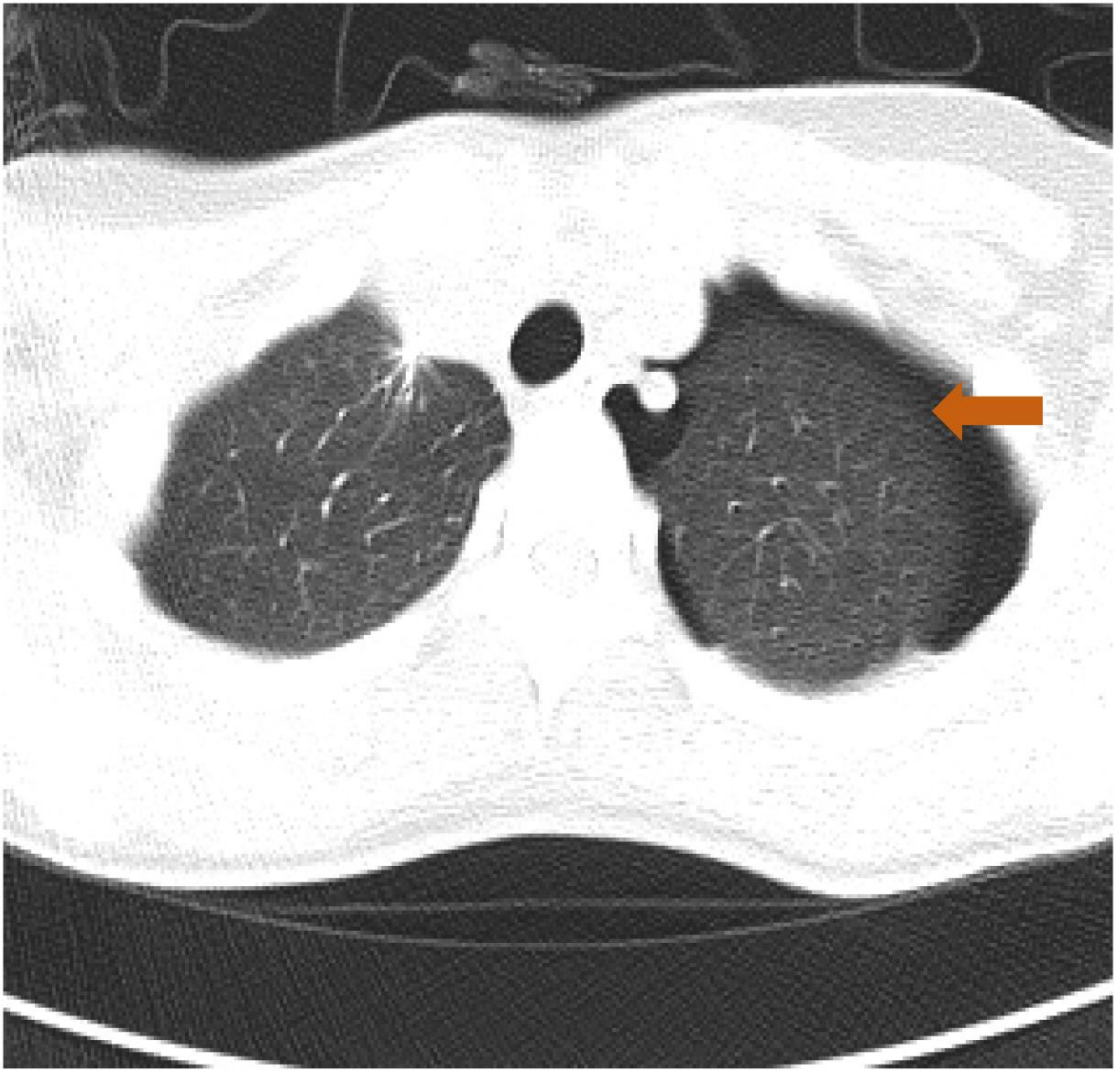
Preoperative chest CT of the second surgery. Left pneumothorax with 50% lung compression.

Ten days after discharge, the patient was readmitted for left spontaneous pneumothorax and received closed thoracic drainage, with temporary symptomatic improvement. However, recurrent left-sided chest pain and pneumothorax occurred 11 days later. Admission chest x-ray confirmed left pneumothorax (Figure 3), and enhanced chest CT revealed approximately 50% left lung compression (Figure 4: Left pneumothorax).

Surgical intervention was recommended for persistent recurrent pneumothorax. Considering the intraoperative mucus hypersecretion and atelectasis complication in the first operation, standardized real-time video-assisted airway surveillance was applied throughout the second surgery for real-time dynamic airway monitoring. Anesthesia induction, maintenance regimens, ventilation parameters, and intraoperative monitoring protocols were strictly consistent with the first operation to eliminate confounding variables.

**Figure 5:**
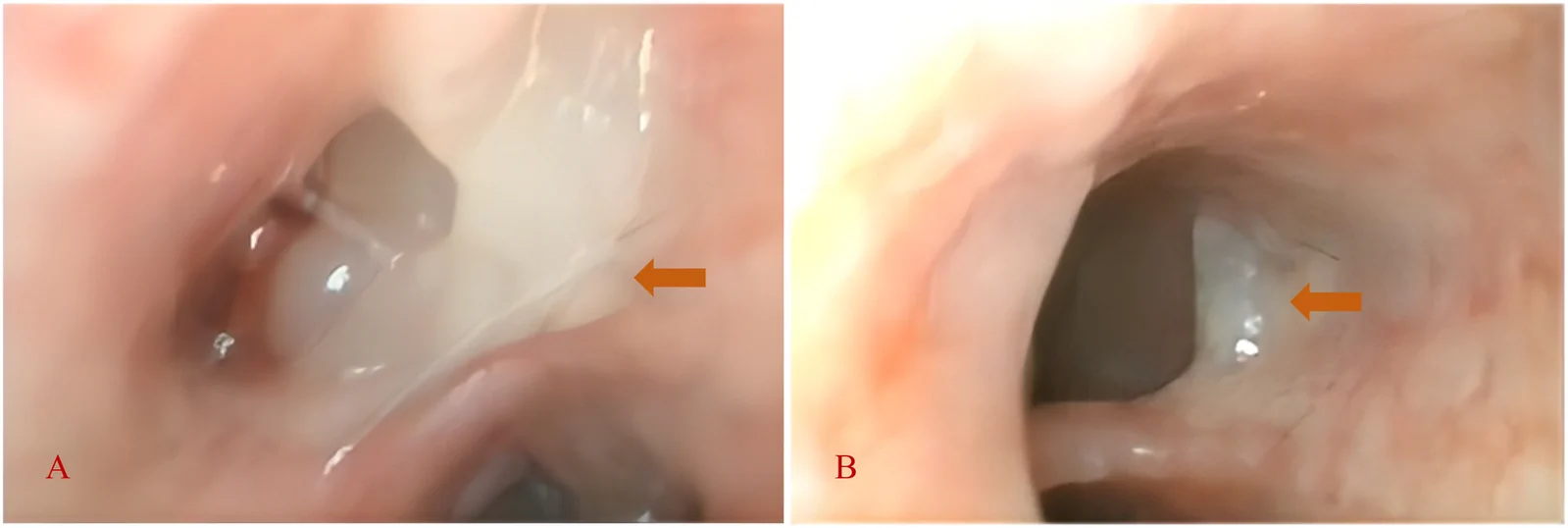
Real-time video-assisted airway surveillance during the second surgery. **(A,B)** Progressive formation of thick viscous mucus in bilateral bronchial trees.

No abnormal mucosal lesions or mucus accumulation were observed on initial bronchoscopic examination after anesthesia induction. However, obvious thick mucus formation in bilateral bronchial trees was detected via real-time airway surveillance approximately 2 h after surgery initiation (Figures 5A,B: Real-time video-assisted airway surveillance mucus formation). Left upper lobe wedge resection was successfully completed, followed by conversion to bilateral lung ventilation and manual lung re-expansion. Consistent with the first operation, partial left upper lobe atelectasis was directly observed under thoracoscopy, which was confirmed to be secondary to segmental bronchial obstruction by massive viscous mucus.

**Figure 6:**
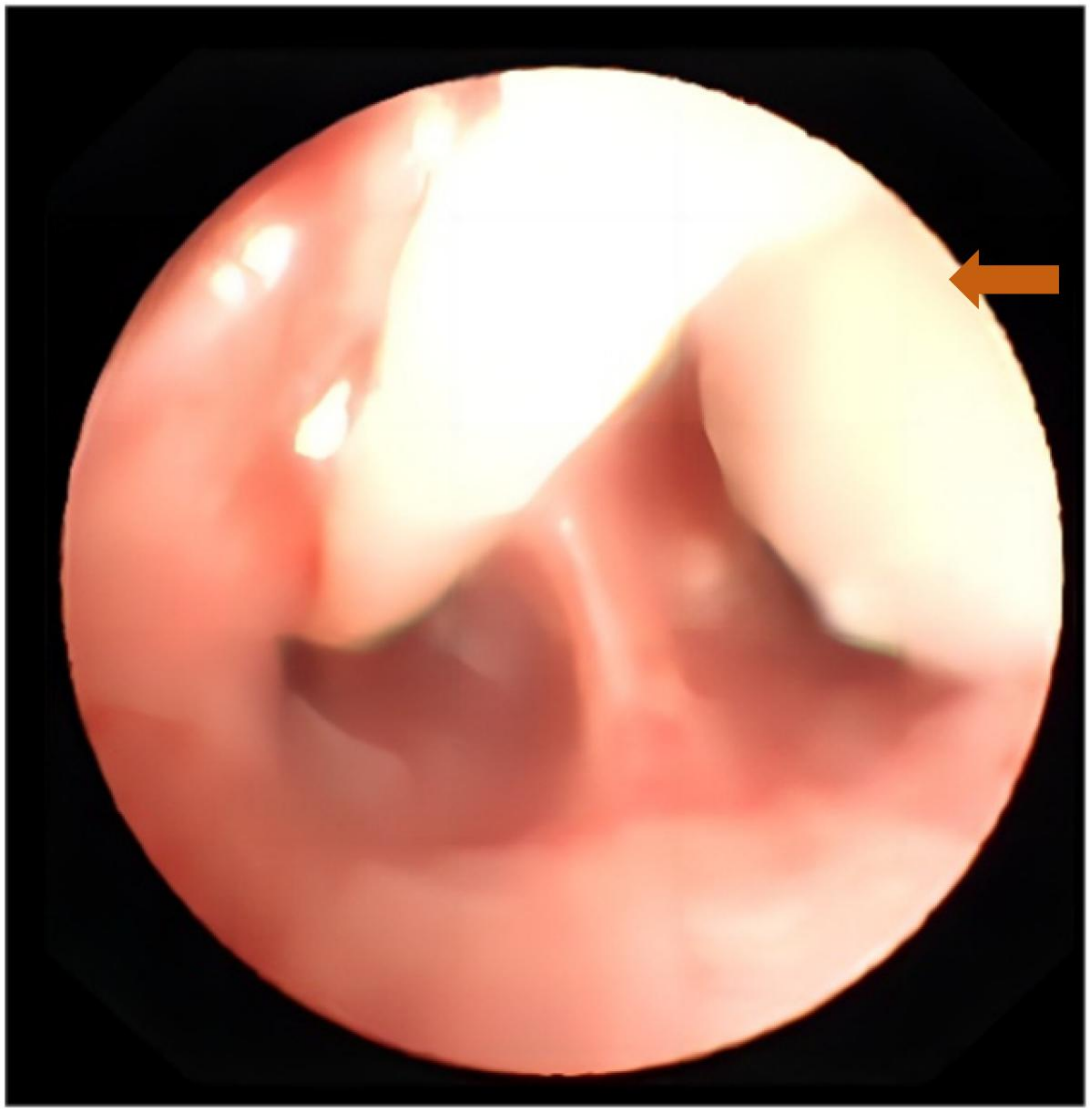
Intraoperative electronic bronchoscopic findings showing extensive gelatinous mucus plugs obstructing segmental bronchi of bilateral lungs.

After obtaining guardian consent, intraoperative pulmonology consultation was performed again. Electronic fiberoptic bronchoscopy revealed extensive mucus plug obstruction involving the left upper lobe lingular and proper segments, right upper lobe segmental orifices, and right lower lobe basal segments (Figure 6: Intraoperative bronchoscopic visualization of thick viscous airway mucus). All obstructed segments were lavaged with 5 mL normal saline per segment, and mucus plugs were completely aspirated and cleared. Satisfactory left lung re-expansion was achieved after lavage. Thoracic drainage was placed, and the operation was completed uneventfully.

**Figure 7:**
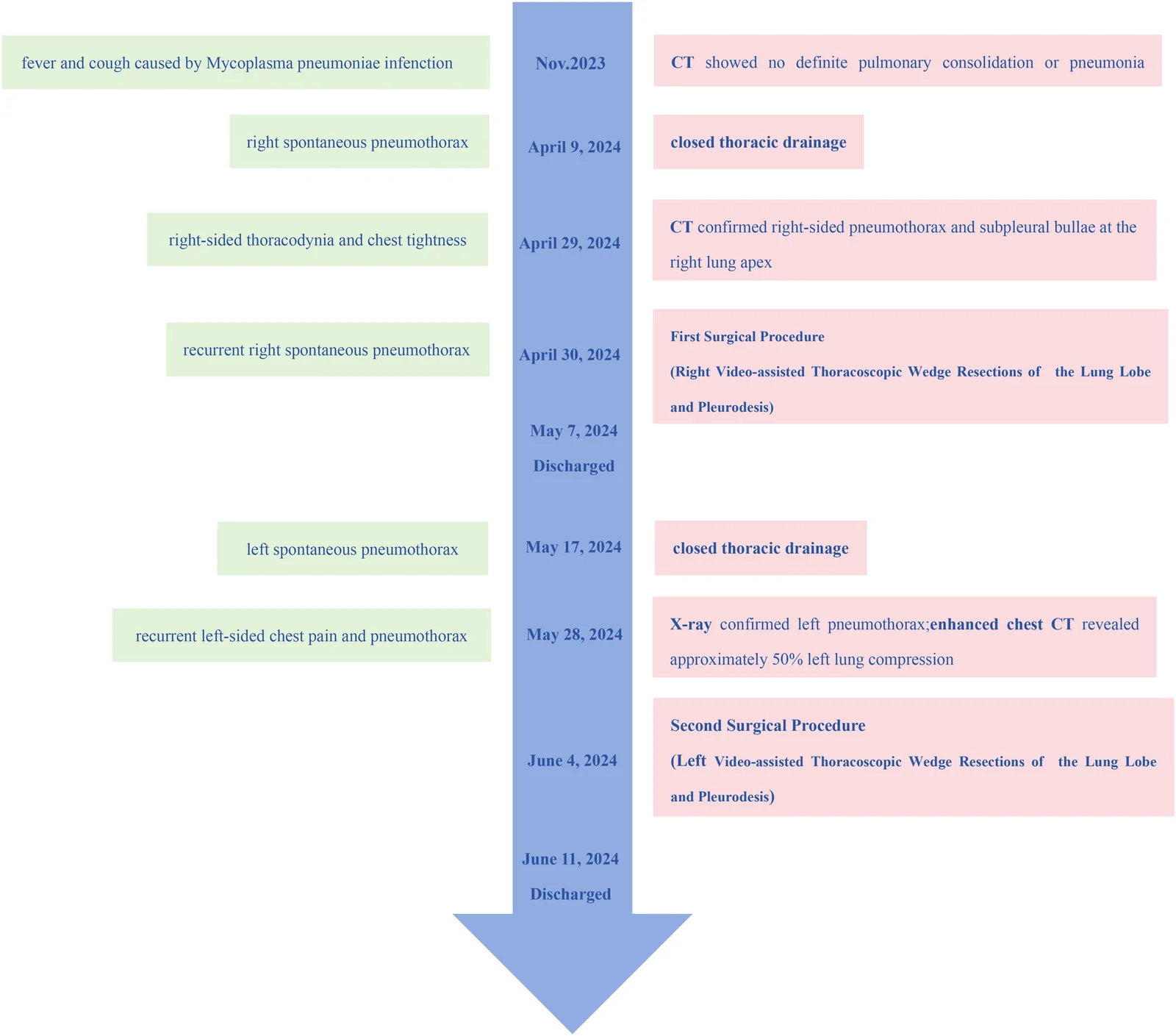
Timeline figure.

The thoracic drainage tube was clamped on postoperative day 4, and follow-up chest imaging showed satisfactory lung expansion. The drainage tube was removed on postoperative day 6, and the patient achieved full clinical recovery with stable vital signs and no respiratory discomfort. She was discharged on postoperative day 7. All treatment procedures for the hospitalized patient are illustrated in (Figure 7).

### Perioperative laboratory and baseline functional data

Retrospective review of laboratory data six months preoperatively confirmed persistent subclinical infection and immune dysregulation: elevated Mycoplasma pneumoniae IgM (1.89 C.O.I) and IgG (194 AU/mL), positive human rhinovirus RNA, decreased NK cell proportion (4.62%), elevated total T lymphocyte ratio (85.5%), and increased interleukin-10 level (10.3 ng/mL). Postoperative first-day re-examination showed persistent positive Mycoplasma pneumoniae serology (IgM 1.33 C.O.I, IgG 135 AU/mL).

## Discussion

Perioperative atelectasis is a multifactorial complication induced by the combined effects of general anesthesia, mechanical ventilation, and surgical trauma, which disrupts alveolar mechanical equilibrium and impairs pulmonary surfactant function (2). OLV during thoracic surgery further exacerbates alveolar collapse and pulmonary inflammatory injury, increasing the risk of atelectasis. To reduce ventilation-induced lung injury during single-lung ventilation, protective lung ventilation strategies were employed for airway management during both intraoperative periods. The present case is unique in that the patient developed identical recurrent intraoperative atelectasis secondary to acquired mucus hypersecretion during two independent sequential unilateral VATS procedures.

Notably, the patient was completely asymptomatic for respiratory disorders before both operations, with no preoperative cough, sputum production, or dyspnea. However, serial laboratory tests confirmed persistent subclinical inflammation induced by prior combined Mycoplasma pneumoniae and human rhinovirus infection. Previous studies have verified that Mycoplasma pneumoniae infection can damage airway epithelial cells, trigger sustained airway inflammatory responses, and induce reactive mucus hypersecretion (3, 4). Human rhinovirus infection further upregulates airway mucus secretion-related genes, promoting excessive mucus production and increased mucus viscosity (5). Although we did not perform intraoperative mucin gene molecular detection to verify MUC5AC/MUC5B expression (a limitation of this case), the consistent intraoperative manifestation of extensive viscous mucus plugs strongly supports infection-induced acquired mucociliary dysfunction, rather than congenital airway disease (6–10).

To eliminate the interference of anesthetic regimens on experimental results, we adopted identical anesthesia induction, maintenance, ventilation parameters, and fluid management strategies in both operations. The only variable was the application of real-time video-assisted airway surveillance in the second surgery, which successfully captured the dynamic process of intraoperative mucus generation and progressive airway obstruction. This adaptive clinical intervention based on prior surgical complications reflects precise and individualized perioperative clinical reasoning.

We speculate that the patient's prior dual viral and mycoplasma infections established a persistent subclinical inflammatory state of the airway, rendering the airway epithelium in a chronically irritated and hyperresponsive state. Surgical stress and OLV-induced lung collapse and inflammatory injury further activated airway inflammatory cascades in both operations, stimulating goblet cell hyperplasia and excessive viscous mucus secretion (11–17). The accumulated gelatinous mucus could not be cleared by routine intraoperative suction, ultimately causing segmental bronchial obstruction and localized atelectasis during lung re-expansion.

The patient's tall-thin body habitus and thoracic structural characteristics are consistent with the high-risk phenotype of adolescent spontaneous pneumothorax (18). However, no radiological or pathological evidence confirmed a direct correlation between pulmonary bullae formation and mucus hypersecretion, indicating that subclinical airway inflammation was the dominant cause of recurrent intraoperative atelectasis.

This case yields important clinical implications for anesthetic management of adolescent patients. Those presenting with a recent history of recurrent respiratory infections and sustained abnormal inflammatory biomarkers require thorough airway evaluation, even without overt respiratory symptoms. Pulmonary function testing serves to assess ventilatory function, while the lung clearance index is capable of detecting subtle impairments in small airway performance and ciliary activity. Specialized assays, including sweat chloride measurement and genetic testing for primary ciliary dyskinesia and cystic fibrosis, help eliminate congenital mucociliary disorders from differential diagnosis (19). Such assessments highlight inherent risks of subclinical airway dysfunction and heightened mucus reactivity during anesthesia. However, due to the limited testing conditions at the hospital where the author works and the fact that both surgeries were performed in emergency settings, a thorough evaluation was not conducted. Preoperative individualized interventions including atomized inhalation therapy can effectively dilute airway mucus, reduce mucosal inflammatory reactivity, and decrease intraoperative obstructive complications. Intraoperatively, real-time video-assisted airway surveillance and flexible bronchoscopic standby are recommended for high-risk patients to achieve early identification and timely clearance of mucus plugs (20), thereby preventing refractory intraoperative atelectasis and reduceing postoperative pulmonary complications.

### Strengths and limitations

#### Strengths

This case report strictly adheres to the CARE guidelines and describes an exceptionally rare and unprecedented complication. Two consecutive surgeries, both managed with identical anesthetic protocols, eliminated patient-specific variability and the influence of anesthetic regimens, thereby providing highly reliable clinical observational data. Based on the available resources at the authors' institution, preoperative laboratory tests, intraoperative monitoring, and postoperative pathological findings collectively confirmed a causal relationship between persistent subclinical inflammation and recurrent intraoperative mucous obstructive atelectasis. The optimized adaptive airway monitoring strategy offers valuable guidance for airway management in high-risk adolescent thoracic anesthesia.

#### Limitations

As a single-case report, this study lacks large-sample prospective validation and cannot establish a definite causal conclusion. No intraoperative molecular testing of airway mucin expression was performed, so the mechanism of mucus hypersecretion is inferred from clinical manifestations and existing literature rather than direct molecular evidence. In addition, long-term postoperative follow-up data are insufficient to evaluate the long-term prognosis of subclinical airway inflammatory dysfunction. Congenital mucociliary disorders, including primary ciliary dyskinesia and cystic fibrosis, were not definitively excluded by specific diagnostic testing such as sweat chloride testing, genetic testing, or specialized ciliary function testing.

### Patient guardian perspective

The patient's parents expressed significant anxiety regarding the child's recurrent pneumothorax and repeated surgical procedures during treatment. They highly recognized the detailed preoperative condition communication and individualized adaptive intraoperative management implemented by the anesthesiology and surgical teams. The real-time airway monitoring and timely bronchoscopic lavage intervention in the second operation effectively avoided potential severe respiratory complications, which greatly relieved parental concerns. The guardians affirmed the value of detailed preoperative assessment of subtle medical history and laboratory abnormalities, and were satisfied with the overall perioperative care and postoperative rehabilitation guidance，and provided full support and understanding by signing informed consent for publication of this case.

## Conclusions

Recurrent intraoperative mucus-obstructive atelectasis in sequential unilateral VATS is an extremely rare perioperative complication. Combined persistent subclinical airway inflammation induced by prior respiratory infections, surgical stress, and OLV-mediated pulmonary inflammatory injury synergistically triggers intraoperative airway mucus hypersecretion, leading to bronchial obstruction and subsequent atelectasis. For adolescent patients undergoing thoracic surgery, comprehensive preoperative evaluation of latent infection history and subclinical inflammatory status, rather than relying solely on symptomatic and radiological evidence, is essential. Individualized intraoperative airway monitoring, precise ventilation and fluid management, and timely bronchoscopic intervention can effectively reduce recurrent perioperative pulmonary complications and improve surgical safety.

## Data Availability

The original contributions presented in the study are included in the article/Supplementary Material, further inquiries can be directed to the corresponding author.
